# Enhancing Digital Reminder Systems for Dementia Care: Exploring Generative AI for Task Verification and Caregiver Support Through Co‐Design

**DOI:** 10.1002/alz70863_110547

**Published:** 2025-12-23

**Authors:** Joy Lai, David Black, Kelly Beaton, Alex Mihailidis

**Affiliations:** ^1^ University of Toronto, Toronto, ON Canada; ^2^ Engagement of People with Lived Experience of Dementia, Toronto, ON Canada

## Abstract

**Background:**

Caregivers of people living with dementia (PLwD) face significant stress, exacerbated by the challenges of verifying task completion despite the use of digital reminder systems. Generative AI, such as GPT, offers a potential solution by improving task verification and supporting caregiver decision‐making. This feasibility study evaluated an AI‐powered task verification system integrated with a digital reminder framework for PLwD. It explored (1) whether GPT can generate high‐quality follow‐up questions tailored to PLwD through few‐shot prompting, (2) the accuracy of the system in flagging concerning responses, and (3) how to balance automation with caregiver control.

**Method:**

An anonymized dataset of 64 reminders was used to simulate interactions between caregivers, PLwD, and the AI system. GPT‐generated follow‐up questions were evaluated for quality with and without contextual information. A flagging mechanism classified responses as High, Medium, or Low concern, with critical categories including mealtime, personal safety, and daily hygiene. Simulated caregiver feedback was incorporated to refine question quality and system adaptability to keep AI‐generated interactions relevant, clear, and minimally intrusive. Additionally, two EPLED (Engagement of People with Lived Experience of Dementia) members were involved in the design process, providing insights into the evaluation methods, few‐shot examples, and usability of the system's responses.

**Result:**

Contextual information and caregiver feedback improved follow‐up question clarity, specificity, and relevance, reducing ambiguity in PLwD responses. The flagging mechanism demonstrated high accuracy, particularly for safety‐critical tasks such as medication and fall prevention reminders, while non‐urgent tasks, such as leisure activities, presented greater subjectivity. Simulated caregiver feedback and EPLED members' input played a crucial role in adapting the system to individual needs and evaluating whether it genuinely reduces caregiver stress or simply redistributes their responsibilities.

**Conclusion:**

This study demonstrates the feasibility of integrating generative AI into dementia care. Context, caregiver input, and EPLED members' perspectives significantly improved task verification and decision support. By enhancing reminder effectiveness and refining caregiver alerts, AI‐assisted verification has the potential to reduce caregiver stress and improve PLwD support. Future research should focus on real‐world validation, user‐centered customization, and scalability to optimize caregiver workload reduction and long‐term adoption in home care settings.

